# Patients' & Healthcare Professionals' Values Regarding True- & False-Positive Diagnosis when Colorectal Cancer Screening by CT Colonography: Discrete Choice Experiment

**DOI:** 10.1371/journal.pone.0080767

**Published:** 2013-12-09

**Authors:** Darren Boone, Susan Mallett, Shihua Zhu, Guiqing Lily Yao, Nichola Bell, Alex Ghanouni, Christian von Wagner, Stuart A. Taylor, Douglas G. Altman, Richard Lilford, Steve Halligan

**Affiliations:** 1 Centre for Medical Imaging, University College London, London, United Kingdom; 2 Department of Primary Care Health Sciences, University of Oxford, Oxford, United Kingdom; 3 Department of Public Health and Epidemiology, Birmingham University, Birmingham, United Kingdom; 4 Faculty of Medicine, University of Southampton, Southampton, United Kingdom; 5 Department of Epidemiology and Public Health, University College London, London, United Kingdom; 6 Centre for Statistics in Medicine, University of Oxford, Oxford, United Kingdom; University Medical Center (UMC) Utrecht, The Netherlands

## Abstract

**Purpose:**

To establish the relative weighting given by patients and healthcare professionals to gains in diagnostic sensitivity versus loss of specificity when using CT colonography (CTC) for colorectal cancer screening.

**Materials and Methods:**

Following ethical approval and informed consent, 75 patients and 50 healthcare professionals undertook a discrete choice experiment in which they chose between “standard” CTC and “enhanced” CTC that raised diagnostic sensitivity 10% for either cancer or polyps in exchange for varying levels of specificity. We established the relative increase in false-positive diagnoses participants traded for an increase in true-positive diagnoses.

**Results:**

Data from 122 participants were analysed. There were 30 (25%) non-traders for the cancer scenario and 20 (16%) for the polyp scenario. For cancer, the 10% gain in sensitivity was traded up to a median 45% (IQR 25 to >85) drop in specificity, equating to 2250 (IQR 1250 to >4250) additional false-positives per additional true-positive cancer, at 0.2% prevalence. For polyps, the figure was 15% (IQR 7.5 to 55), equating to 6 (IQR 3 to 22) additional false-positives per additional true-positive polyp, at 25% prevalence. Tipping points were significantly higher for patients than professionals for both cancer (85 vs 25, p<0.001) and polyps (55 vs 15, p<0.001). Patients were willing to pay significantly more for increased sensitivity for cancer (p = 0.021).

**Conclusion:**

When screening for colorectal cancer, patients and professionals believe gains in true-positive diagnoses are worth much more than the negative consequences of a corresponding rise in false-positives. Evaluation of screening tests should account for this.

## Introduction

Understanding diagnostic test performance is essential for evidence-based practice [1], [2], particularly for screening where risks and benefits are balanced finely. No screening test is 100% sensitive and the consequence is readily understood; false-negative tests will delay or prevent cure. Specificity is important for screening because most people are disease-free. A false-positive test means healthy individuals may undergo invasive procedures causing anxiety, morbidity, and even mortality [3]. Tests that increase the proportion of people with disease who test true-positive (increase sensitivity) usually simultaneously increase the proportion of people without disease who test false-positive (diminish specificity). For example, computer-aided-detection (CAD) [4], digital imaging [5], and shorter screening intervals [6] all increase mammographic sensitivity for breast cancer but decrease specificity.

When comparing two diagnostic tests, interpretation is sometimes difficult if one has high sensitivity and the other high specificity. A combined measure of sensitivity and specificity facilitates interpretation; examples include net-benefit or the area under the receiver-operator characteristic curve (ROC AUC) [7]–[11]. An advantage of net-benefit measures is that they can incorporate relative values for gains in true-positive diagnoses versus costs of false-negative diagnoses, whereas ROC AUC cannot. However, few studies have quantified these costs and those that have suggest they are valued very differently by patients; one study found women would accept 500 false-positive mammograms to avoid a single missed cancer [12]. While qualitative research suggests that attendees value sensitivity over specificity when screening for colorectal cancer [13], [14] this has not been quantified. Ignoring these preferences may underestimate test benefit. For example, the Medicaid/Medicare decision to not reimburse CT colonography (CTC) did not consider that screenees may still value gains in sensitivity despite diminished specificity [15]. To clarify this issue we established the relative weighting given by patients and healthcare professionals to additional true-positive diagnoses compared to additional false-positive diagnoses (i.e. gains in sensitivity versus loss of specificity) when using CTC for colorectal cancer screening.

## Methods

### Ethics Statement

Ethics committee approval was granted by the local institutional ethical review board of University College Hospitals London; all participants gave written informed consent.

### Design

We designed and conducted a discrete choice experiment (DCE) [16]–[18], according to recent guidelines [18]. In particular, patients may value sensitivity so highly that even small changes can mask the influence of other attributes [18]. Also, specificity is conceptually challenging, with patients often unaware that false-positive diagnoses can occur [13]. Therefore, to simplify decision-making we used a ‘probability equivalence’ design to establish attitudes to sensitivity and specificity alone, without the influence of other attributes. We presented sensitivity and specificity in terms of differing numbers of true-positive and false-positive diagnoses by imaging. A hypothetical “enhanced” CTC screening test was presented against “standard” CTC and participants noted their preference between the two. Sensitivity and specificity for cancer for “standard” CTC was 85% and 95% respectively and 80% and 85% for polyps ≥6 mm. “Enhanced” CTC raised sensitivity for cancer to 95%, equivalent to detecting one additional cancer per 5000 screenees (cancer prevalence 0.2%) [19], [20]. “Enhanced” CTC raised sensitivity for polyps to 90%, equivalent to detecting 125 additional people with polyps per 5000 (polyp prevalence 25%) [21]. We aimed to raise sensitivity by 10% while avoiding a perfect test, which is unrealistic. Specificity of “enhanced” CTC was dropped in increments to 10% for cancer and 20% for polyps (Table 1) across the scenarios. Such extremely low specificity is unlikely in real practice but necessary to calculate “tipping points”, i.e. the level at which an individual is willing to “trade” one attribute for the other. In the present case, the tipping point was the maximum reduction in specificity that participants were prepared to trade for a 10% absolute (vs relative) increase in sensitivity.

**Table 1:** Overview of attributes and levels presented in cancer and polyp discrete choice experiments.

Cancer question number	“standard” CTC		“Enhanced” CTC				
	Sensitivity for cancer (%)	Specificity for cancer (%)	Sensitivity for cancer (%)	Specificity for cancer (%)	Additional true positive detections per 5000 screening examinations	Additional false positive detections per 5000 screening examinations	FP tipping point (specificity: Standard CTC minus enhanced CTC) (%)
1c*	85	95	95	95	1	0	0
2c*	85	95	95	95	1	0	0
3c	85	95	95	90	1	250	5
4c	85	95	95	80	1	750	15
5c	85	95	95	70	1	1250	25
6c	85	95	95	50	1	2250	45
7c	85	95	95	40	1	2750	55
8c	85	95	95	30	1	3250	65
9c	85	95	95	20	1	3750	75
10c***	85	95	95	10	1	4250	85

Polyp question number	“standard” CTC		“Enhanced” CTC				
	Sensitivity for polyps (%)	Specificity for polyps (%)	Sensitivity for polyps (%)	Specificity for polyps (%)	Additional true positive detections per 5000 screening examinations	Additional false positive detections per 5000 screening examinations	FP tipping point (specificity: standard CTC minus enhanced CTC) (%)
1p*	80	85	90	90	125	−250	−5
2p*	80	85	90	85	125	0	0
3p**	80	85	90	80	125	250	5
4p**	80	85	90	80	125	250	5
5p	80	85	90	70	125	750	15
6p	80	85	90	60	125	1250	25
7p	80	85	90	50	125	1750	35
8p	80	85	90	40	125	2250	45
9p	80	85	90	30	125	2750	55
10p***	80	85	90	20	125	3250	65

^*^ Questions both favour enhanced CTC for both sensitivity and specificity. Respondents choosing standard CTC were considered to have misunderstood the task.

^**^ Questions are identical to test for internal consistency.

^***^ Participants choosing enhanced CTC in response to question 10 were considered potential non-traders; i.e.. they considered detection of a single additional cancer worth 4250 additional colonoscopies.

Because DCEs are difficult to comprehend, especially via postal questionnaires [22], we used an interviewer-led design for patients, which clarifies understanding and permits qualitative exploration afterwards, especially with non-traders [23] (a “non-trader” is a participant who will not trade their preferred attribute at any cost. With respect to the present study, this would usually represent an individual who would accept any value of diminished specificity in order to achieve 10% increased sensitivity). A multimedia laptop presentation of colorectal cancer screening by colonoscopy and CTC was given, including information on survival benefit, that early detection was not always curative [24], and that that false-positive CTC caused unnecessary colonoscopy. For clarity, only the most serious colonoscopic complication was presented, perforation in 1∶500 [25], [26]. Because inconsistent framing may introduce bias [27], both absolute and relative risks were displayed textually and graphically. Participants were asked to assume they were average risk for cancer/polyps and that polypectomy would reduce lifetime disease-specific mortality by 25% [28].

A random scenario was repeated to test consistency. A scenario with one option unquestionably superior for both sensitivity and specificity identified “irrational” responders. Finally, we incorporated “willingness-to-pay” (WTP) assessment: Standard CTC was pitched against CTC with sensitivity raised by 10% but with identical specificity. Participants were asked how much, if anything, they would pay for this.

### Pilot

10 volunteers were piloted to confirm comprehensibility and inform sample size [29]. While understanding attributes and levels, some did not trade (i.e. they judged the lowest specificity acceptable). We therefore reprogrammed additional “stress-slides” (automatically triggered by responses accepting the lowest specificity for enhanced CTC), reinforcing potential harms, to assess whether heuristic bias anchored their decision. Seemingly irrational responses declined on repeat piloting of the same volunteers. Also, participants had been confused by considering cancer and polyps simultaneously, so the final survey presented separate cancer and polyp DCEs sequentially, each consisting of 10 scenarios.

### Recruitment

We recruited consecutive consenting patients of screening age (>55 years), scheduled for non-cancer outpatient ultrasound/plain-radiographic investigations at a teaching hospital, identified via booking systems. Information/consent forms were mailed and responders interviewed on their appointment day. To avoid bias we excluded respondents with a personal history of, or being investigated for, bowel cancer [30]. All participants were offered a £10 gift voucher.

To investigate any attitudinal difference between patients and healthcare professionals, we recruited radiologists, gastroenterologists, surgeons, nurse-specialists, and radiographers who requested, performed, or interpreted colorectal imaging. To facilitate recruitment, healthcare professionals could complete the DCE online since we considered they were familiar with the concepts presented. Otherwise, a radiologist or clinical psychologist conducted DCEs, with scenarios presented in random order within the two DCEs. All participants were asked their age, ethnicity, education, and household income bracket.

### Analysis

**Figure 1:**
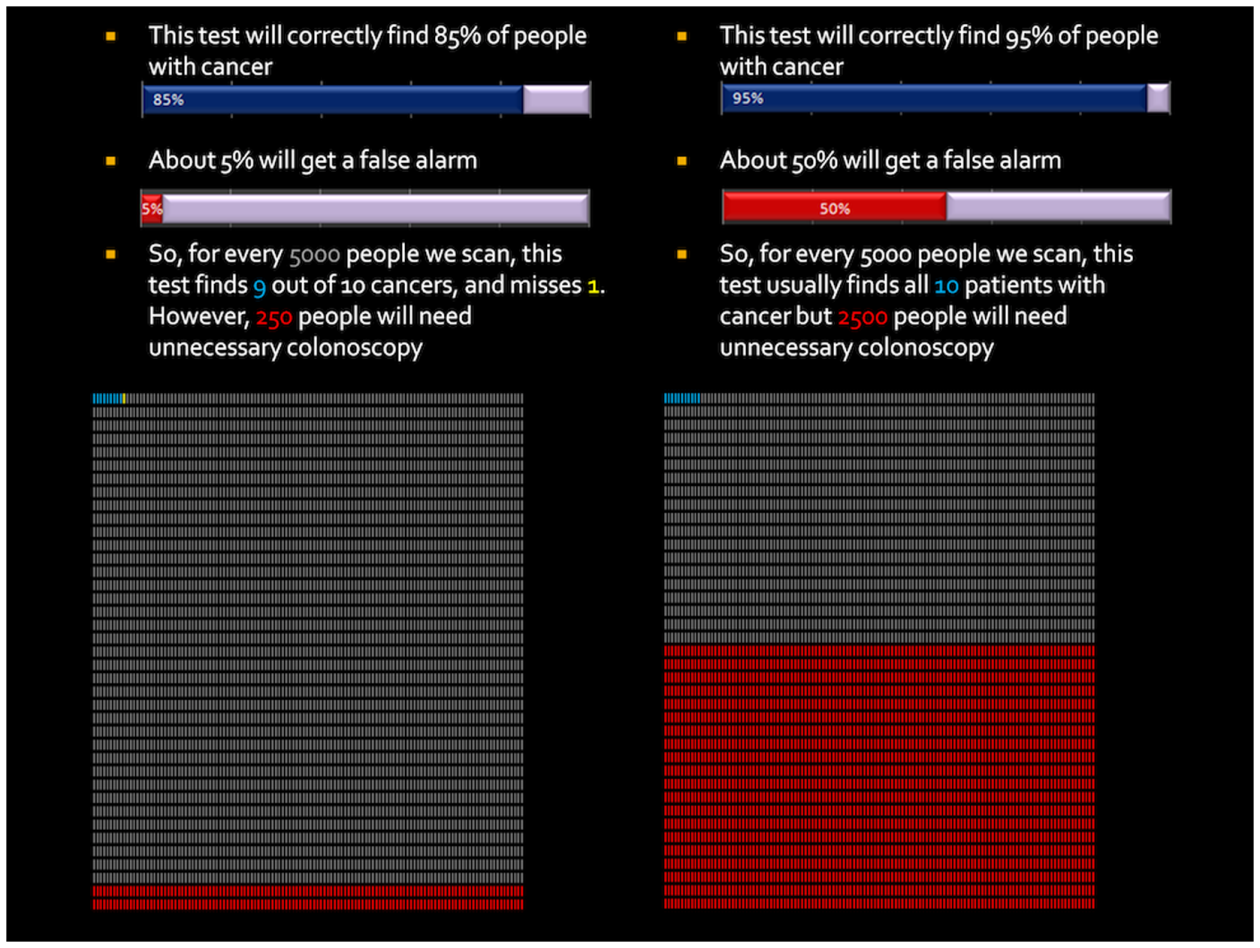
Example question from the cancer detection scenario. Each tally mark represents one of 5000 potential outcomes for a patient undergoing screening: True positive (blue), false negative (yellow), true negative (white), or false positive (red). Participants were informed that if they were to undertake the test in question, their odds of receiving any of the outcomes were represented by the chance of picking any of these tally-marks at random “like roulette”. Data are also represented numerically using both relative and absolute percentages. This scenario corresponds to the ‘tipping point’ for patients and professional respondents: On average, participants favoured the enhanced test (test B) in view of its additional sensitivity up to, but not beyond, this level of additional false positives.

Our primary outcome was the reduction in specificity participants were willing to “trade” for 10% absolute (vs relative) increase in sensitivity. We defined the “tipping-point” as the highest increase in false-positive rate (FPR; 1-specificity) above baseline at which participants perceived the benefits of increased sensitivity were outweighed by potential harms. In the pilot this was 45% for cancer (i.e. participants allowed specificity to fall from 95% to 50% on average). To determine the median tipping point ±5% at two-sided alpha 0.05 and 90% power required 96 participants (*N* = 4σ^2^ *z_crit_* ^2^/*D* ^2^ where *D* = 0.10, *p* = 0.45, *z*_crit_ = 1.960, σ = 0.25) [31]. We pre-specified a secondary outcome comparing patients and professionals, for which 88 participants were required for 90% power to detect 15% difference. Because our pilot suggested non-normality we recruited a further 15% [31]. The stress-slides were triggered by participants preferring “enhanced” CTC despite increasing FPR by 85% for cancer and 65% for polyps. The highest tipping-point was taken if they traded subsequently; others were deemed persistent non-traders. Because participants were presented simultaneously with sensitivity, specificity, pictorial descriptions of changes and numerical information on the absolute increase in false-positives compared to increase in true-positives (Figure 1), we framed our results in terms of false-positive vs true-positive diagnoses, as this is most easily understood.

The median tipping-point was calculated for cancer and polyps, for patients/healthcare professionals combined and separately. Because patient and professional numbers differed we used values from 1999 bootstrap estimates of median and IQRs, where samples included equal numbers (n = 50) of each group, therefore weighting patients and professionals equally. At the tipping point, the change in specificity equivalent to a 10% change in sensitivity was converted into a change in the absolute relative numbers of false-positive and true-positive diagnoses using the equation for net benefit [11], [32]:

net benefit = 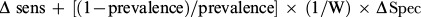


Where Δ sens = 10%, and Δspec = median tipping point, and W is the relative weighting (the maximum number of additional false-positives traded per additional cancer or polyp detected). Prevalence was assigned 0.2% for cancer, 25% for polyps [19]–[21].

**Figure 2:**
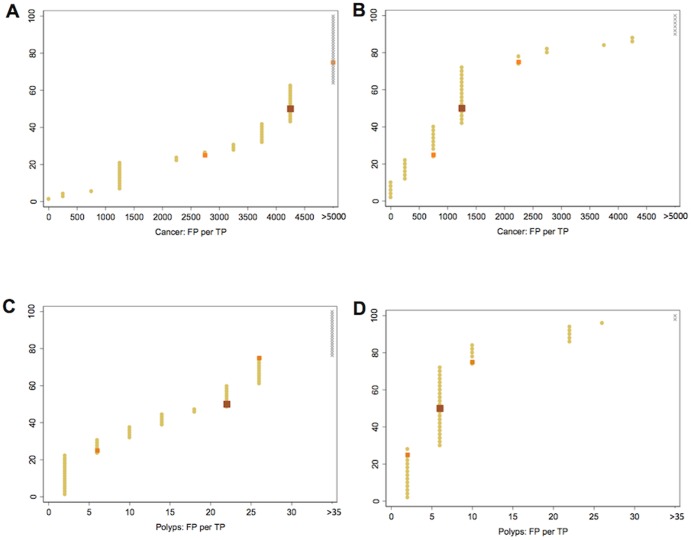
Cumulative graph of participants' tipping points for trading absolute numbers of true-positive versus false-positive diagnoses. Each yellow dot shows an individual participant's trading point. Grey symbols indicate values assigned for participants who refused to trade. Brown dot shows median value representing “an average participant”. Orange dots show 25 and 75 percentage points. Graphs are shown separately as follows: A; Patients, cancer scenario (n = 72). B; Professionals, cancer scenario (n = 50). C; Patients, polyp scanario (n = 72). D; Professionals, polyp scanario (n = 50).

Tipping points were compared between patients interviewed by each researcher. Tipping points were highly non-normal so were summarised by medians and interquartile ranges (IQR 25% to 50%); the median can be interpreted as corresponding to an average participant. For tipping points and relative weighting of false-positive to true-positive, all non-traders were treated as requiring higher FP values than offered (Figure 2: grey values). The Mann-Whitney U test statistic and Wilcoxon signed rank sum test were used for unpaired and paired comparison, respectively (Stata V11.0, Stata Corporation, College Station, Texas).

## Results

112 consecutive patients and 62 professionals were invited. 75 patients and 50 healthcare professionals participated, a response of 67% and 81% respectively (Table 2). Three patients could not complete the DCE leaving 122 for analysis (two medical professionals gave partial responses). DB interviewed 53, NB interviewed 48; 21 responses were online. Compared to professionals, patients were older, discontinued education earlier, and had lower household income (Table 2).

**Table 2:** Demographic characteristics and household annual income of patient and professional participants, including non-traders.

Characteristic	Patients (n = 72)*	Professionals (n = 50)**	Total (n = 122)
**Gender**			
Female	49 (68)	24 (48)	73 (60)
Male	23 (32)	26 (52)	49 (40)
**Age (year)**			
25–34	0 (0)	26 (52)	26 (21)
35–54	0 (00)	23 (46)	26 (21)
55–59	18 (25)	1 (2)	16 (13)
60–69	40 (56)	0 (0)	40 (33)
70–79	14 (19)	0 (0)	14 (12)
**Ethnicity**			
White	49 (70)	33 (66)	82 (67)
Other	23 (32)	17 (34)	40 (33)
**Household income/GBP/year**			
<10000	3 (4)	0 (0)	3 (3)
10001–20000	14 (19)	0 (0)	14 (11)
20001–30000	19 (26)	3 (6)	22 (18)
30001–40000	10 (14)	9 (18)	19 (15)
>40000	4 (6)	32 (64)	36 (30)
**Declined to answer**	22 (31)	6 (12)	28 (23)

Data are number (percentage).

^*^ Of the original 75 patients accrued, 3 discontinued the survey without providing any consistent, logical responses. Qualitative exploration by the interviewer revealed they did not understand the process so data were not included.

^**^ Comprising 5 gastroenterologists, 5 radiologists, 5 colorectal surgeons, 10 Specialist registrars in these specialities, 5 bowel cancer screening nurses and 20 CT radiographers.

### Non-traders

For cancer detection 30 (25%; 27 patients, 3 professionals) participants were non-traders, 20 (16%; 18 patients, 2 professionals) of who were also non-traders for polyps. Non-traders were significantly more likely to be patients (27[38%] vs 3[6%]); p<0.001), were significantly older (median age 64.5 vs 44.5; p<0.001), and were less educated than traders (15% vs 2% with no formal qualifications; p<0.001). There was no significant difference in gender (59% vs 61% female; p = 0.56) or ethnicity (30% vs 33% non-white; p = 0.57). Considering patients alone, non-traders (n = 27) were older (median age 66.8 vs 60.1; p = 0.001), less affluent (median household income GBP10001-20000 vs. GBP20001-£30000 per annum; p = 0.03. GBP = Great British Pound,  = 1.2 Euros and 1.6 US Dollars at current exchange rates) and less qualified (median school-leaving age 16 vs. 18yrs; p = 0.02) than traders (n = 45). For cancer and polyps respectively, 34% (16/47) and 35% (11/31) participants who were initially unwilling, subsequently traded following the stress-slides.

### Cancer

Overall, the median tipping-point for cancer detection occurred at 45% drop in specificity (IQR 25 to >85%; Table 3). Thus, on average, a 45% drop in specificity was considered the maximal fall acceptable in exchange for 10% increased sensitivity. At population prevalence of 0.2%, this equates to 2250 (IQR 1250 to >4250) additional false-positive diagnoses per additional true-positive cancer. The average number of additional false-positives per additional true-positive detection was significantly higher for patients (median 4250 (IQR 2750 to >4250) than professionals (median 1250, IQR 750 to 2250, p<0.001), i.e. the average patient perceived a greater number of false-positives acceptable to gain an additional true-positive.

**Table 3:** Tipping points and relative weighting for cancer and polyp detection scenarios calculated for patients, professionals, and all participants combined (FP = false-positive diagnosis, TP = true-positive diagnosis).

	Tipping point (FP tipping point: max change in specificity acceptable for a 10% gain in sensitivity)		Relative weighting FP to TP (Average number of additional FP per additional TP detection)	
	Median	IQR	Median	IQR
**Patients**				
Polyp	55	15 to 65	22	6 to 26
Cancer	85	55 to >85	4250	2750 to >4250
**Professionals**				
Polyp	15	5 to 25	6	2 to 10
Cancer	25	15 to 45	1250	750 to 2250
**Combined**				
Polyp	15	7.5 to 55	6	3 to 22
Cancer	45	25 to >85	2250	1250 to >4250

### Polyps

Overall, the median tipping-point for polyp detection was 15% (IQR 7.5 to 55; Table 3). Thus, on average, a 15% drop in specificity was considered the maximal fall acceptable in exchange for 10% increased sensitivity. At population prevalence of 25%, this equates to 6 (IQR 3 to 22) additional false-positive diagnoses per additional true-positive polyp. Again, the median number of additional false-positives per additional true-positive was significantly higher for patients (55, IQR 15 to 65) than professionals (15, IQR 5 to 25. p<0.001).

For patients and professionals combined, the average number of additional false-positives traded per additional true-positive detection was significantly higher for cancer than polyps (45 vs 15; p<0.001), indicating that larger falls in specificity were perceived acceptable when testing for cancer.

There was no significant difference in overall median tipping point elicited by the radiologist or psychologist, (p = 0.57) nor between medical professionals' data obtained face-to-face vs online (p = 0.59).

### Willingness-to-pay

Three quarters of participants were willing to give a price range they would be willing to pay for a test with sensitivity raised by 10% but no loss of specificity. Median WTP was significantly higher for cancer than polyps: 201–500GBP (IQR 101–200GBP to 501–1000 GBP) vs. 101–200 GBP (IQR 51–100 to 201–500 GBP), p<0.001, indicating participants felt cancer detection was worth more than polyp detection. There was no significant difference in WTP for polyp detection when patients and professionals were compared (p = 0.97) but patients' WTP was significantly higher than professionals' for cancer detection: median 201–500 GBP (IQR 101–200 GBP to 201–500GBP) vs median 101–200 GBP (IQR 51–100 GBP to 201–500 GBP, p = 0.036). Moreover, median household income was significantly lower for patients than professionals (20001–25000GBP vs >40000GBP; p = 0.021, Table 4), indicating that patient's values were particularly strongly held from a relative perspective. Most participants (27 of 32 participants) who declined to answer WTP, declined to answer for both polyps and cancers. Participants who declined gave, on average, higher values of false-positives per additional true-positive diagnosis.

**Table 4:** Patient and professionals' willingness to pay (WTP) for a 0.10 (10%) increase in test sensitivity without any reduction in specificity, for detection of cancer or clinically significant polyps.

WTP/GBP	Polyp detection					
	Patients (72)		Professionals (50)		Total (122)	
	n	%	n	%	n	%
<50	9	12	8	16	17	14
51–100	10	14	8	16	18	15
101–200	15	21	14	28	29	24
201–500	4	6	10	20	14	11
501–1000	10	14	4	8	14	11
>1000	0	0	0	0	0	0
Declined to answer	24	33	6	12	30	25

WTP/GBP	Cancer detection					
	Patients (72)		Professionals (50)		Total (122)	
	n	%	n	%	n	%
<50	5	7	5	10	10	8
51–100	3	4	7	14	10	8
101–200	10	14	12	24	22	18
201–500	14	20	9	18	23	19
501–1000	11	15	6	12	17	14
>1000	8	11	3	6	11	9
Declined to answer	21	29	8	16	29	24

GBP = Great British Pounds.

## Discussion

In relation to screening for colorectal cancer and polyps, patients and healthcare professionals both valued gains in diagnostic sensitivity over and above corresponding loss of specificity. On average, 2250 additional false-positives were considered worth trading for a single additional true-positive diagnosis of cancer and 6 additional false-positives for an additional true-positive diagnosis of a polyp. Our findings are similar to those from a study of mammography that found women willing to trade 500 false-positive mammograms (and their consequences) in order to diagnose a single additional cancer that would otherwise have been missed [12]. While it is known that patients value sensitivity over specificity for colorectal cancer screening [33], [34], we could find no data that quantified this for a radiological test. Our interest was stimulated by studies of CAD for CTC, which increases sensitivity but at the cost of reduced specificity, sometimes significantly [35]–[38]. However, the potential clinical consequences of missed cancer (death) are not equivalent to those of false-positive diagnosis (unnecessary colonoscopy); our findings confirm that both patients and healthcare professionals believe this. It is therefore important that analysis of research studies of diagnostic tests take account of this asymmetry. Diagnostic tests can be compared using net-benefit measures, which incorporate relative weightings for different clinical costs [11], [39]. By contrast statistical measures such as ROC AUC cannot assign different utilities to gains in sensitivity versus falls in specificity and so could find a new test of no value when both patients and professionals might think otherwise.

We used a discrete choice experiment, a relatively novel methodology for establishing preferences [40]. Traditionally, preferences are elicited via ranking [41], with test attributes considered in isolation. Results are therefore predictable: Patients and professionals favour tests that are sensitive, specific, inexpensive, and non-invasive. However, this does not reflect the trade-offs demanded by real practice. DCEs are increasingly advocated by researchers because respondents indicate preferences between different test characteristics, which more accurately reflects real-world choices [16]–[18], [41]–[43]. Because DCEs are complex, we delivered most experiments face-to-face to facilitate understanding and participation, which can increase the generalizability of results. Accordingly, most participants gave complete, consistent, meaningful responses. While interviewer bias is possible, we found no significant difference between responses obtained from the psychologist or radiologist. Further, there was no significant difference in responses obtained face-to-face or online.

To simplify and focus the cognitive task, we compared just two attributes, increase in true-positive and false-positive diagnoses by imaging (also expressed by sensitivity and specificity). In order to create an “enhanced” test that inflated sensitivity for cancer to 95% (perfect sensitivity would be unrealistic) we used a baseline sensitivity of 85% for standard CTC, which is likely an underestimate. However, in this type of experiment, the relative weighting given to attributes across different scenarios is key, not the absolute difference between them. Using this design we elicited the relative importance that participants placed on gains in sensitivity versus loss of specificity.

Although both groups valued gains in sensitivity over and above corresponding loss of specificity, this finding was stronger for patients. Healthcare professionals, especially those who are medically qualified, will have a deeper understanding of the pros and cons of diagnostic testing; as noted earlier, some patients do not understand that false-positive diagnosis is possible [13]. Patients were older, discontinued education earlier, and had approximately half the annual household income of health professionals, yet patients ascribed a monetary value to enhanced sensitivity that was approximately twice that of professionals, demonstrating they consider sensitivity exceptionally important.

If statistical analyses must account for discrepant weightings for sensitivity and sensitivity, a particularly interesting question is whose weightings should be used? Some will argue that healthcare professionals are the best option, notably medically qualified clinicians because they request tests, have the deepest understanding of pros and cons, and thus have the broadest and most informed perspective overall. Others will argue that society ultimately undergoes and pays for diagnostic testing, and so patients' perspectives are most appropriate. This issue warrants further research.

Our study has limitations. As noted previously, DCEs are challenging for participants [44], requiring motivation, literacy, and numeracy, which may introduce selection bias [23]. We attempted to reduce this effect by using an interviewer rather than a postal questionnaire. Although we had power for our primary endpoint, larger and/or different samples will better investigate differences between subcategories of patients and healthcare professionals. Because we believed that we should not ignore particularly strongly held beliefs, we included non-traders via calculating median values; our estimates are therefore an underestimate. WTP estimates are also likely underestimates because of reluctance to state income. We followed guidelines for best practice of DCE studies [18] but suggest that strategies for design and analysis need further investigation [45], [46]. Common to all hypothetical scenarios, subjects' actions in real life may not mirror those expressed in a DCE. Finally, the weightings we derived are specific to colorectal cancer screening. However, we believe they are likely to be similar to other scenarios that involve diagnosis of cancer [12].

In summary, via discrete choice experiment we found that both patients and healthcare professionals believe gains in diagnostic sensitivity are worth more than the perceived negative consequences of a corresponding loss of specificity, when considering colorectal cancer screening. Gains in sensitivity over loss of specificity were valued more highly for cancer detection (vs polyps) and by patients rather than healthcare professionals. These effects should influence the evaluation of new screening tests.
